# ﻿*Papiliomycessinensis* (Clavicipitaceae) and *Paraisariapseudoarcta* (Ophiocordycipitaceae), two new species parasitizing Lepidopteran insects from southwestern China

**DOI:** 10.3897/mycokeys.117.150376

**Published:** 2025-05-15

**Authors:** Hui Chen, Shabana Bibi, Ling Tao, Xiangchun Shen, Jun Zhao, Yueming Sun, Qirui Li, Dexiang Tang, Yao Wang

**Affiliations:** 1 State Key Laboratory of Discovery and Utilization of Functional Components in Traditional Chinese Medicine & School of Pharmaceutical Sciences, Guizhou Medical University, Guian New District, Guizhou 561113, China; 2 The High Efficacy Application of Natural Medicinal Resources Engineering Center of Guizhou Province, Guizhou Medical University, Guian New District, Guizhou 561113, China; 3 Department of Biosciences, Shifa Tameer-e-Millat University, Islamabad 44000, Pakistan; 4 Guizhou Tongde Pharmaceutical Co., Ltd., Tongren, Guizhou, 554300, China

**Keywords:** *Cordyceps*, Entomopathogenic fungi, new species, phylogenetics, taxonomy

## Abstract

*Cordycepssensu lato* species are highly important for medicinal purposes and functional food nutrients. Two new species belonging to *Cordycepssensu lato* are introduced, i.e., *Papiliomycessinensis* and *Paraisariapseudoarcta*. To comprehensively describe the significance of these two species, morphological data were supplemented with phylogenetic analyses based on six loci (nrSSU, ITS, nrLSU, *tef-1α*, *rpb1*, and *rpb2*). Phylogenetically, *Pap.sinensis* is most closely related to *Pap.albostromaticus* and *Pap.shibinensis*, yet it can be distinguished from them by its larger stromata (51.3–85.7 × 3.1–3.5 vs. 37.0–58.0 × 2.5–3.0) and longer phialides (10.1–26.9 × 0.9–3.3 vs. 9.8–24.3 × 1.5–3.1). *Paraisariapseudoarcta* is phylogenetically sister to *Par.arcta*. The longer stromata (43–51 vs. 16) and larger secondary ascospores (5.6–8.3 × 1.7–3.1 vs. 2.6–4.2 × 0.5–1.3) in *Par.pseudoarcta* are characteristics that distinguish the two species. A thorough morphological description and phylogenetic analysis of *Pap.sinensis* and *Par.pseudoarcta* were provided. In addition, taxonomic misconceptions of *Par.gracilis* (Ophiocordycipitaceae) were corrected.

## ﻿Introduction

The latest classification of *Cordycepssensu lato* (*s. l.*) places it in the family Clavici­pitaceae *s. l.*, which is characterized by filiform ascospores that often disarticulate into secondary ascospores, thickened ascus apices, and cylindrical asci (Mains 1958; Kobayasi 1982; Rossman et al. 1999; Hywel-Jones 2002; Sung et al. 2007a). Given its wide host range and abundance of species, this genus represents the most diverse group within the Clavicipitaceae*s. l.* Four cordycipitoid families are now recognized in the order Hypocreales: Clavicipitaceae, Cordycipitaceae, Ophiocordycipitaceae, and Polycephalomycetaceae. Together, these four families contain more than 1,300 cordycipitoid species and at least 42 genera (Chaverri et al. 2005; Luangsa-ard et al. 2005, 2017; Sung et al. 2007a; Zare and Gams 2007; Johnson et al. 2009; Kepler et al. 2012a, 2012b, 2014; Quandt et al. 2014; Spatafora et al. 2015; Tsang et al. 2016; Mongkolsamrit et al. 2019; Xiao et al. 2023).

Similarly, revisions have occurred in the classification of other genera within Hypocreales. One significant genus of entomopathogenic fungi frequently found in various terrestrial habitats is *Metarhizium* (Kepler et al. 2014; Brunner-Mendoza et al. 2019). Initially, *Metarhizium* species were classified based on morphological traits, leading to the identification of species such as *M.anisopliae*, *M.flavoviride*, and *M.album* (Tulloch 1976; Driver et al. 2000). However, accurate identification has often been hindered by morphological convergence, particularly in distinguishing cryptic species. The advent of molecular techniques has transformed fungal taxonomy by providing robust tools for species delimitation (Johannes et al. 2011; Rehner et al. 2011; Huzefa et al. 2017). Multilocus phylogenetic studies, including those by Sung et al. (2007a, b), Kepler et al. (2012a, 2014), and Mongkolsamrit et al. (2020), have significantly improved the resolution in differentiating closely related species. Mongkolsamrit et al. (2020) revised *Metarhizium*, accepting 66 species, including 21 within the *M.anisopliae* complex and 13 within the *M.flavoviride* complex. This revision also led to the establishment of six new genera—*Keithomyces*, *Marquandomyces*, *Papiliomyces*, *Purpureomyces*, *Sungia*, and *Yosiokobayasia*—highlighting the dynamic and evolving nature of fungal taxonomy. Among these newly erected genera, *Papiliomyces* was established by Mongkolsamrit et al. (2020) in the family Clavicipitaceae (Hypocreales, Sordariomycetes) based on phylogenetic analyses, initially accommodating two species: *Papiliomycesliangshanensis* (collected from Nepal) and *Papiliomycesshibinensis* (collected from China). The genus was defined by morphological and multilocus phylogenetic data, distinguishing it from *Metarhizium*. However, subsequent studies questioned the taxonomic placement of the type species, *P.liangshanensis*, which was later transferred to *Ophiocordyceps* based on phylogenetic evidence (Wang et al. 2021). This revision underscores the critical role of molecular systematics in fungal taxonomy and highlights the ongoing refinement of *Papiliomyces* classification. The genus name is derived from the Latin word *papilio*, meaning “butterfly” or “moth” (Mongkolsamrit et al. 2020). The primary teleomorphic characteristics of *Papiliomyces* include solitary to multiple stromata, perithecia ranging from superficial to completely immersed, cylindrical asci, and ascospores that either remain entire with septation or fragment into cylindrical part-spores (Kepler et al. 2012a, b; Mongkolsamrit et al. 2020; Wang et al. 2021). These unique morphological features set *Papiliomyces* apart from other genera in the Clavicipitaceae.

Petch (1931) established the genus *Ophiocordyceps*, with *O.blattae* as the type species, in contrast to *Papiliomyces*. The ascospores of *Ophiocordyceps* remain intact and become septate after discharge (i.e., non-disarticulating ascospores), and the asci possess faint apical caps. Kobayasi (1941) later classified *Ophiocordyceps* as a subgenus of Cordyceps. Subsequently, Sung et al. (2007b) separated *Ophiocordyceps* from Cordyceps based on molecular phylogenetic analyses and provided a comprehensive re-description of the genus. *Ophiocordyceps* exhibits a wide range of morphological characteristics, including stromata that may be fibrous, hard, pliant, or wiry, with colors ranging from dark to light. The perithecia may be immersed and arranged in either an ordinal or oblique pattern. Additionally, *Ophiocordyceps* displays two distinct ascospore morphologies: entire ascospores and those that fragment into part-spores either within the ascus or after ejection, as reported by Ban et al. (2015), Sanjuan et al. (2015), Araújo et al. (2018), Khonsanit et al. (2018), and Luangsa-ard et al. (2018). Based on multigene phylogenies presented in the referenced literature (Quandt et al. 2014; Sanjuan et al. 2015; Araújo et al. 2018), *Ophiocordyceps* is characterized by a remarkable diversity of asexual morphs, including but not limited to *Hirsutella*, *Hymenostilbe*, *Paraisaria*, *Sorosporella*, *Stilbella*, and *Syngliocladium*.

The asexually typified genus *Paraisaria* was proposed by Samson and Brady (1983), with *Par.dubia* (syn. *Isariadubia* Delacr.) as the type species. *Paraisariadubia* is a well-established species occurring on the larvae of *Hepialus* (Lepidoptera) and is also associated with *Ophiocordycepsgracilis* (syn. *Cordycepsgracilis* (Grev.) Durieu & Mont.). Another notable species, *Par.gracilioides*, corresponds to *O.gracilioides* (syn. *Cordycepsgracilioides* Kobayasi), which is recognized as its sexual morph. *Paraisaria* is characterized by white, loose synnemata composed of verticillately branched, sympodially proliferating polyphialides with swollen bases and narrowly cylindrical to fusiform conidia formed in slimy heads. The sexual morphs are distinguished by unique morphological features, including a globose fertile structure at the terminal end of the stroma, immersed perithecia, and the presence of part-spores within the ascus and after ascospore discharge (Samson and Brady 1983).

However, it is important to note that the hosts of *Cordycepss. l.* primarily belong to several arthropod orders, with Coleoptera and Lepidoptera being the two most prominent (Shrestha et al. 2016). Of the approximately 200 *Cordyceps* species parasitizing these orders, 92 species have been documented with identified host species. Among Coleopteran hosts, Scarabaeidae and Elateridae are the primary families. Similarly, among Lepidopteran hosts, Hepialidae is the largest host family.

For the past two years, our efforts have been exerted in the investigation of cordycipitoid fungi, especially in southwestern China. In this study, two unknown species of *Cordycepss. l.* attacking Lepidopteran larvae were collected from Guizhou and Yunnan provinces, southwestern China. These two species were different from all other *Cordycepss. l.* species in morphology and combined multi-gene phylogeny analyses. Hence, they are recognized as new species.

## ﻿Materials and methods

### ﻿Specimen collection and fungus isolation

Most of the specimens used in this investigation were gathered from Yunnan Province, China, while a few specimens were gathered from Guizhou Province’s Yuntai Mountain Scenic Area. During field investigations, specimens were photographed, and relevant data were recorded. After being kept at low temperatures (-4 °C) in plastic containers, the samples were brought to the lab for identification. The specimens were thereafter kept in the Guizhou Medical University Herbarium (GMB). Samples were first soaked for five minutes in a 30% hydrogen peroxide solution before being washed twice with sterile distilled water. Sterile paper was used to blot away any surface dampness (Sun et al. 2022). The tissue was moved to potato glucose agar (PDA) plates after the treated samples were aseptically sliced to remove the epidermis. For storage, purified fungal cultures were either moved to PDA slants at 4 °C or kept in an incubator at 25 °C (Wang et al. 2020). Living cultures were deposited at the Guizhou Medical University Culture Collection (GMBC).

### ﻿Morphological characteristic observations

A Nikon SMZ745T stereomicroscope (Tokyo, Japan) was used to study the specimen’s macroscopic features, including its size, color, shape, length, stroma, and fertile head. A Nikon ECLIPSE Ni compound microscope (Nikon, Japan) was used to take pictures of the samples using a Canon EOS 700D digital camera. Measurements were made using the Tarosoft (R) Image Frame Work (v.0.9.7). The colony was grown on PDA plates in an incubator set at 25 °C for 14 days. After that, the colony was measured and photographed using the equipment and tools mentioned before.

### ﻿DNA extraction, PCR amplification, and sequencing

The samples were put in a sterile centrifuge tube and processed until they were completely pulverized using sterile fine rods. The genomic DNA purification kit (Qiagen GmbH, Hilden, Germany) was used to isolate genomic DNA in accordance with the manufacturer’s instructions. At -20 °C, the pure DNA was kept. 1 µL of DNA extract, 1 µL of each forward and reverse primer (10 µM each), 9.5 µL of ddH_2_O, and 12.5 µL of 2 × Taq PCR Master Mix (2 × Taq Master Mix with dye, TIANGEN, China) made up the 25 µL PCR reaction mixture. The primer combination NS1 and NS4 was used to amplify the nuclear ribosomal small subunit (nrSSU) (White et al. 1990). The primer combination ITS4 and ITS5 was used to amplify the nuclear ribosomal internal transcribed spacer region (ITS) (White et al. 1990). The primer pair 28F and 28R was used to amplify the nuclear ribosomal large subunit (nrLSU) (Stephen and Samuels 1994). The primer combination TEF-F and TEF-R was used to amplify the translation elongation factor 1α (*tef-1α*) (Bischoff et al. 2006; Sung et al. 2007a). The primer pairs CRPB1-5’F and CRPB1-5’R, as well as fRPB2-5F and fRPB2-7cR, were used to amplify the largest and second-largest subunits of RNA polymerase II (*rpb1* and *rpb2*) (Liu et al. 1999; Castlebury et al. 2004; Bischoff et al. 2006) (Table 1). The PCR assays of the six genes were conducted as described by Wang et al. (2015). An automatic sequence analyzer (BGI Co., Ltd., Shenzhen, China) was used to sequence the PCR products after they had been separated by electrophoresis in 1.0% agarose gels and purified using the Gel Band Purification Kit (Bio Teke Co., Ltd., Beijing, China).

**Table 1.** The primer information of each gene fragment used for DNA amplification in this study.

Gene	Primer name	Primer sequence (5'-3')	Reference
nrSSU	NS1	GTAGTCATATGCTTGTCTC	White et al. (1990)
	NS4	CTTCCGTCAATTCCTTTAAG	
nrLSU	28F	ACCCGCTGAACTTAAGC	Stephen and Samuels (1994)
	28R	ATCCTGAGGGAAACTTCG	
*tef-1α*	TEF-F	GCTCCYGGHCAYCGTGAYTTYAT	Bischoff et al. (2006); Sung et al. (2007)
	TEF-R	ATGACACCRACRGCRACRGTYTG	
*rpb1*	RPB1-5’F	CAYCCWGGYTTYATCAAGAA	Castlebury et al. (2004); Bischoff et al. (2006)
	RPB1-5’R	CCNGCDATNTCRTTRTCCATRTA	
*rpb2*	fFPB2-5F	GAYGAYMGWGATCAYTTYGG	Liu et al. (1999)
	fRPB2-7cR	CCCATRGCTTGYTTRCCCAT	
ITS	ITS4	TCCTCCGCTTATTGATATGC	White et al. (1990)
	ITS5	GGAAGTAAAAGTCGTAACAAGG	

### ﻿Phylogenetic analyses

Sequences of the six loci (nrSSU, ITS, nrLSU, *tef-1α*, *rpb1*, and *rpb2*) were retrieved from GenBank. Table 2 contains the taxonomy details and GenBank accession numbers for these sequences. MAFFT v.7 (http://mafft.cbrc.jp/alignment/server/) and MEGA 7.0.26 were used to align the sequences (Tamura et al. 2013). Where required, the aligned sequences were subsequently manually adjusted. MEGA 7.0.26 was used to concatenate the aligned sequences of nrSSU, ITS, nrLSU, *tef-1α*, *rpb1*, and *rpb2* into a single dataset. Maximum likelihood (ML) and Bayesian inference (BI) were used for phylogenetic studies. GTR + FO + G was selected as the optimal model for ML analysis, and 1000 rapid bootstrap replicates were performed on the dataset. ML phylogenetic analyses were conducted in RAxML 7.0.3 (Stamatakis et al. 2008). Additional ML analyses were performed using IQ-TREE v. 2.1.3 with ultrafast bootstrapping for the estimation of branch support (Minh et al. 2020). jModelTest v.2.1.4 was used to determine the best-fit model for BI analysis (Darriba et al. 2012). The analysis used the following models: GTR + I + G for partitions of nrSSU, ITS, nrLSU, and *tef-1α* and GTR + I for partitions of *rpb1* and *rpb2*. Bayesian analysis was conducted using MrBayes v.3.2.7a for five million generations (Ronquist et al. 2012). *Samsoniellaasiatica*YFCC 869 and *Samsoniellahepiali*YFCC 868 were designated as the outgroup taxa for the analysis. The phylogenetic tree was viewed and edited using FigTree V.1.4.2. Furthermore, ML analyses (IQ-TREE) were performed separately for each locus: nrSSU, ITS, nrLSU, *tef-1α*, *rpb1*, and *rpb2*.

**Table 2.** Relevant species information and GenBank accession numbers for phylogenetic research in this study.

Species	Voucher / information	Host / Substrate	GenBank Accession Number						Reference
			ITS	nrSSU	nrLSU	*tef-1α*	*rpb1*	*rpb2*	
*Conoideocrellahuteorostata*	NHJ 12516	Lepidoptera	JN049860	EF468994	EF468849	EF468800	EF468905	EF468946	Sung et al. (2007a)
*Conoideocrellatenuis*	NHJ 6293	Lepidopteran pupa	JN049862	EU369112	EU369044	EU369029	EU369068	EU369087	Sung et al. (2007a)
*Drechmeriabalanoides*	CBS 250.82^T^	Nematoda	NA	AF339588	AF339539	DQ522342	DQ522388	DQ522442	Sung et al. (2001); Spatafora et al. (2007)
*Drechmeriacampanulata*	IMI 356051^T^	*Panagrolaimus*	NA	AF339592	AF339543	NA	NA	NA	Sung et al. (2001)
*Drechmeriapanacis*	CBS 142798^T^	*Panaxnotoginseng*	NA	MF588890	MF588897	MF614144	NA	NA	Yu et al. (2018)
*Drechmeriazeospora*	CBS 335.80^T^	*Panagrolaimus*	NA	AF339589	AF339540	EF469062	EF469091	EF469109	Sung et al. (2001); Sung et al. (2007a)
*Harposporiumharposporiferum*	ARSEF 5472^T^	Arthropod	NA	AF339569	AF339519	DQ118747	DQ127238	NA	Sung et al. (2001); Chaverri et al. (2005)
*Harposporiumanguillulae*	ARSEF 5407	Soil	NA	NA	AY636080	NA	NA	NA	Chaverri et al. (2005)
*Keithomycesacicularis*	JCM 33284^T^	Soil	LC435734	LC435738	LC435741	LC462188	NA	NA	Iwasaki et al. (2019)
*Keithomycesacicularis*	JCM 33285	Soil	LC463198	LC435739	LC435742	LC462189	NA	NA	Iwasaki et al. (2019)
*Keithomycescarneus*	CBS 399.59	Soil	MT078887	NA	MH869445	NA	NA	NA	Sung et al. (2007b)
*Keithomycescarneus*	CBS 239.32^T^	Sand dune	AY624171	EF468988	EF468843	EF468789	EF468894	EF468938	Sung et al. (2007b)
*Keithomycesneogunnii*	GZUHSB13050302^T^	Lepidoptera larva	KU729716	KU729722	NA	KU729727	KU729732	NA	Wen et al. (2017)
*Marquandomycesmarquandii*	CBS 182.27^T^	Soil	AY624193	EF468990	EF468845	EF468793	EF468899	EF468942	Sung et al. (2007b)
*Marquandomycesmarquandii*	CBS 128893	*Hygrocybevirginea*	MH865143	NA	MH876582	NA	NA	NA	Sung et al. (2007b)
*Marquandomyces* sp.	CBS 127132	Soil	MT078882	MT078872	MT078857	MT078849	MT078865	MT078922	Sung et al. (2007b)
*Marquandomyces* sp.	CBS 129413	Soil	MT561567	MT078874	MT078859	MT078851	MT078867	NA	Sung et al. (2007b)
*Metacordycepsliangshanensis*	EFCC 1452	Lepidoptera pupa	NA	EF468962	EF468815	EF468756	NA	NA	Sung et al. (2007b)
*Metacordycepsliangshanensis*	EFCC 1523	Lepidoptera pupa	NA	EF468961	EF468814	EF468755	NA	EF468918	Sung et al. (2007b)
*Metapochoniabulbillosa*	CBS 145.70^T^	*Piceaabies*	AJ292410	AF339591	AF339542	EF468796	EF468902	EF468943	Sung et al. (2007b); Sung et al. (2001)
*Metapochoniabulbillosa*	JCM 18596	*Piceaabies*	NA	AB758252	NA	AB758460	AB758663	AB758690	Sung et al. (2007b)
*Metapochoniagoniodes*	CBS 891.72^T^	Nematoda	AJ292409	AF339599	MH872319	DQ522354	DQ522401	DQ522458	Spatafora et al. (2007)
*Metapochoniasuchlasporia*	CBS 251.83^T^	Nematode egg	MH861580	NA	MH873311	KJ398790	KJ398601	KJ398697	Kepler et al. (2014); Vu et al. (2019)
*Metapochoniasuchlasporia*	CBS 248.83^T^	Nematode egg	MH861579	NA	MH873310	KJ398789	KJ398600	KJ398696	Kepler et al. (2014); Vu et al. (2019)
*Metapochoniarubescens*	CBS 464.88^T^	Nematode egg	MH862138	AF339615	MH873830	EF468797	EF468903	EF468944	Sung et al. (2007)
*Metarhiziumalbum*	ARSEF 2082	Hemiptera	AY375446	DQ522560	DQ518775	DQ522352	DQ522398	DQ522452	Kepler et al. (2012a)
Metarhiziumcf.album	ARSEF 2179	Hemiptera	NA	NA	NA	KJ398807	KJ398618	KJ398716	Kepler et al. (2012a)
*Metarhiziumanisopliae*	CBS 130.71^T^	*Avenasativa*	MT078884	MT078868	MT078853	MT078845	MT078861	MT078918	Gisbert (1993)
*Metarhiziumbrunneum*	ARSEF 2107^T^	Coleoptera	KC178691	NA	MH868397	EU248855	EU248907	EU248935	Bischoff et al. (2009)
*Metarhiziumchaiyaphumense*	BCC 19020	Hemiptera: Cicadidae	HQ165694	HQ165654	HQ165716	HQ165675	HQ165737	HQ165635	Luangsa-ard et al. (2017)
*Metarhiziumchaiyaphumense*	BCC 78198^T^	Hemiptera: Cicadidae	NA	KX369596	KX369593	KX369592	KX369594	KX369595	Luangsa-ard et al. (2017)
*Metarhiziumflavoviride*	ARSEF 2025	Coleoptera	AF138269	NA	NA	KJ398804	KJ398614	KJ398712	Kepler et al. (2014)
*Metarhiziumflavoviride*	CBS 218.56^T^	Coleoptera	MH857590	NA	MH869139	KJ398787	KJ398598	KJ398694	Kepler et al. (2014); Vu et al. (2019)
*Metarhiziumgaoligongense*	CCTCCM2016588^T^	Soil	KY087808	KY087812	KY087816	KY087820	KY087824	KY087826	Chen et al. (2018)
*Metarhiziumguizhouense*	ARSEF 6238	Lepidoptera	NA	NA	NA	EU248857	EU248909	EU248937	Bischoff et al. (2009)
*Metarhiziumguizhouense*	CBS 258.90	Lepidoptera	HQ331448	NA	NA	EU248862	EU248914	EU248942	Bischoff et al. (2009)
*Metarhiziumglobosum*	ARSEF 2596^T^	Lepidoptera	HQ331459	NA	NA	EU248846	EU248898	EU248926	Bischoff et al. (2009)
*Ophiocordycepskimflemingiae*	SC 30	Hymenoptera	NA	KX713629	KX713622	KX713699	KX713727	NA	Araújo et al. (2018)
*Ophiocordycepskonnoana*	EFCC 7315	Coleoptera	NA	EF468959	NA	EF468753	EF468861	EF468916	Sung et al. (2007a)
*Ophiocordycepslongissima*	TNS F18448	Hemiptera	NA	KJ878925	KJ878892	KJ878971	KJ879005	NA	Quandt et al. (2014)
*Ophiocordycepsmonticola*	BPI 634610	Orthoptera	OQ709246	NA	NA	NA	NA	NA	Tehan et al. (2023)
*Ophiocordycepsnigrella*	EFCC 9247	Coleoptera	JN049853	EF468963	EF468818	EF468758	EF468866	EF468920	Sung et al. (2007a)
*Ophiocordycepspulvinata*	TNSF 30044	Hymenoptera	NA	GU904208	NA	GU904209	GU904210	NA	Kepler et al. (2011)
*Ophiocordycepsravenelii*	OSC 151914	Coleoptera	NA	KJ878932	NA	KJ878978	KJ879012	KJ878950	Quandt et al. (2014)
*Ophiocordycepssinensis*	EFCC 7287	Lepidoptera	JN049854	EF468971	EF468827	EF468767	EF468874	EF468924	Quandt et al. (2014)
*Ophiocordycepsstylophora*	OSC 111000	Coleoptera	JN049828	DQ522552	DQ518766	DQ522337	DQ522382	DQ522433	Quandt et al. (2014)
*Ophiocordycepsvariabilis*	OSC 111003	Diptera	NA	EF468985	EF468839	EF468779	EF468885	EF468933	Sung et al. (2007a)
*Ophiocordycepsvariabilis*	ARSEF 5365	Diptera	NA	DQ522555	DQ518769	DQ522340	DQ522386	DQ522437	Spatafora et al. (2007)
*Papiliomycesalbastromata*	YHH 23070027	Hepialidae	OR770519	OR770494	OR770504	PP479838	PP203269	PP479841	Chen et al. (2024)
*Papiliomycesalbastromata*	YHH 2307003	Hepialidae	OR770518	OR770493	OR770503	PP479837	PP203268	PP479840	Chen et al. (2024)
*Papiliomycesalbastromata*	YFCC 23079297	Hepialidae	OR775109	OR775107	OR775108	PP479839	PP203270	PP479842	Chen et al. (2024)
*Papiliomyceslongiclavatus*	YC 20061403^T^	Lepidoptera larva	MZ702080	MZ702112	MZ702101	MZ955880	MZ955876	MZ955872	Zhang et al. (2023)
*Papiliomyceslongiclavatus*	YC 20061407	Lepidoptera larva	MZ702082	MZ702114	MZ702103	MZ955882	NA	NA	Zhang et al. (2023)
*Papiliomycesshibinensis*	GZUHSB13050311^T^	Lepidoptera	KR153585	KR153588	NA	KR153589	KR153590	NA	Wen et al. (2015)
***Papiliomycessinensis***	**GMB 3053**	***Napialus* larva**	**PQ636502**	**PQ636499**	**PQ636505**	**PQ660654**	**PQ660657**	**PQ660660**	**This study**
***Papiliomycessinensis***	**GMBC 3053^T^**	***Napialus* larva**	**PQ636503**	**PQ636500**	**PQ636506**	**PQ660655**	**PQ660658**	**PQ660661**	**This study**
***Papiliomycessinensis***	**GMBC 3054**	***Napialus* larva**	**PQ636504**	**PQ636501**	**PQ636507**	**PQ660656**	**PQ660659**	**PQ660662**	**This study**
*Papiliomycespuniceum*	BUM 838^T^	Lepidoptera	OM955149	OM951244	OM951249	NA	OM988194	OM988189	Chen et al. (2023)
*Papiliomycespuniceum*	BUM 1214	Lepidoptera	OM955150	OM951245	OM951250	OM988198	OM988195	OM988190	Chen et al. (2023)
*Paraisariaalba*	HKAS 102484^T^	Orthoptera	MN947219	MN943843	MN943839	MN929085	MN929078	MN929082	Wei et al. (2021)
*Paraisariaamazonica*	HUA 186143	Orthoptera	NA	KJ917562	KJ917571	KM411989	KP212902	KM411982	Sanjuan et al. (2015)
*Paraisariaamazonica*	HUA 186113	Orthoptera	NA	KJ917566	KJ917572	NA	KP212903	KM411980	Sanjuan et al. (2015)
*Paraisariaarcta*	HKAS 102553^T^	Lepidoptera	MN947221	MN943845	MN943841	MN929087	MN929080	NA	Wei et al. (2021)
*Paraisariaarcta*	HKAS 102552	Lepidoptera	MN947220	MN943844	MN943840	MN929086	MN929079	MN929083	Wei et al. (2021)
*Paraisariablattarioides*	HUA 186093	Blattodea	NA	KJ917559	KJ917570	KM411992	KP212910	NA	Sanjuan et al. (2015)
*Paraisariablattarioides*	HUA 186108^T^	Blattodea	NA	KJ917558	KJ917569	NA	KP212912	KM411984	Sanjuan et al. (2015)
*Paraisariacascadensis*	OSC-M-052010	Orthoptera	OQ709237	OQ800918	OQ708931	OR199814	OR199828	OR199838	Tehan et al. (2023)
*Paraisariacascadensis*	OSC-M-052017	Orthoptera	OQ709240	OQ800921	OQ708934	OR199817	OR199831	NA	Tehan et al. (2023)
*Paraisariacoenomyia*	NBRC 106964	Diptera	AB968397	AB968385	AB968413	AB968571	NA	AB968533	Ban et al. (2015)
*Paraisariacoenomyia*	NBRC 108993^T^	Diptera	AB968396	AB968384	AB968412	AB968570	NA	AB968532	Ban et al. (2015)
*Paraisariagracilioides*	HUA 186095	Coleoptera	NA	NA	NA	KM411994	KP212914	NA	Sanjuan et al. (2015)
*Paraisariagracilioides*	HUA 186092	Coleoptera	NA	NA	KJ130992	NA	KP212915	NA	Sanjuan et al. (2015)
*Paraisariagracilis*	EFCC 3101	Lepidoptera	NA	EF468955	EF468810	EF468750	EF468858	EF468913	Sung et al. (2007a)
*Paraisariagracilis*	EFCC 8572	Lepidoptera	JN049851	EF468956	EF468811	EF468751	EF468859	EF468912	Sung et al. (2007a)
*Paraisariagracilis*	OSC 151906	Lepidoptera	NA	KJ878923	KJ878890	KJ878969	NA	NA	Quandt et al. (2014)
***Paraisariagracilis***	**GMBC 3066**	Lepidoptera pupa	**PQ787761**	**PQ785776**	**PQ785779**	**PQ789222**	**PQ789225**	**PQ789228**	**This study**
*Paraisariaheteropoda*	EFCC 10125	Hemiptera	JN049852	EF468957	EF468812	EF468752	EF468860	EF468914	Sung et al. (2007a)
*Paraisariaheteropoda*	NBRC 100643	Hemiptera	NA	JN941719	JN941422	AB968595	JN992453	AB968556	Ban et al. (2015)
*Paraisariainsignis*	OSC 164134	Coleoptera	OQ709231	OQ800911	OQ708924	OR199807	OR199822	NA	Tehan et al. (2023)
*Paraisariainsignis*	OSC 164135	Coleoptera	OQ709232	OQ800912	OQ708925	OR199808	OR199823	NA	Tehan et al. (2023)
*Paraisariaorthopterorum*	BBC 88305	Orthoptera	MH754742	NA	MK332583	MK214080	MK214084	NA	Mongkolsamrit et al. (2019)
*Paraisariaorthopterorum*	TBRC 9710^T^	Orthoptera	MH754743	NA	MK332582	MK214081	MK214085	NA	Mongkolsamrit et al. (2019)
*Paraisariaphuwiangensis*	TBRC 9709^T^	Coleoptera	MK192015	NA	MK192057	MK214082	MK214086	NA	Mongkolsamrit et al. (2019)
***Paraisariapseudoarcta***	**GMBC 3064^T^**	Lepidoptera pupa	**PQ787759**	**PQ785774**	**PQ785777**	**PQ789220**	**PQ789223**	**PQ789226**	**This study**
***Paraisariapseudoarcta***	**GMBC 3065**	Lepidoptera pupa	**PQ787760**	**PQ785775**	**PQ785778**	**PQ789221**	**PQ789224**	**PQ789227**	**This study**
*Paraisariapseudoheteropoda*	OSC-M-052007	Hemiptera	OQ709235	OQ800916	OQ708929	OR199812	OR199826	OR199837	Tehan et al. (2023)
*Paraisariapseudoheteropoda*	OSC-M-052022	Hemiptera	OQ709245	OQ800925	OQ708939	OR199821	OR199835	OR199841	Tehan et al. (2023)
*Paraisariarosea*	HKAS 102546^T^	Coleoptera	MN947222	MN943846	MN943842	MN929088	MN929081	MN929084	Wei et al. (2021)
*Paraisariatettigonia*	GZUHCS14062709^T^	Orthoptera	KT345954	KT345955	NA	KT375440	KT375441	NA	Wang et al. (2016)
*Pochoniaboninensis*	JCM 18597	Soil	AB709858	AB758255	AB709831	AB758463	AB758666	AB758693	Nonaka et al. (2013)
*Pochoniaglobispora*	CBS 203.86^T^	Soil	DQ516079	NA	MH873631	NA	NA	NA	Zare and Gams (2007)
*Pochoniachlamydosporia*	CBS 504.66^T^	Soil	AJ292398	AF339593	AF339544	EF469069	EF469098	EF469120	Sung et al. (2007a); Sung et al. (2001)
*Pochoniachlamydosporia*	CBS 101244	Mollusca	JN049821	DQ522544	DQ518758	DQ522327	DQ522372	DQ522424	Spatafora et al. (2007)
*Purpureocilliumatypicolum*	CBS 744.73	*Atypuskarschi*	NA	EF468987	EF468841	EF468786	EF468892	NA	Sung et al. (2007a)
*Purpureocilliumatypicolum*	OSC 151901	*Atypuskarschi*	NA	KJ878914	KJ878880	KJ878961	KJ878994	NA	Quandt et al. (2014)
*Purpureocilliumlilacinum*	CBS 284.36^T^	Hemiptera	NA	AY526475	FR775484	EF468792	EF468898	EF468941	Johnson et al. (2009)
*Purpureocilliumlilacinum*	NHJ 3497	Hemiptera	NA	EU369096	EU369033	EU369014	EU369053	EU369074	Johnson et al. (2009)
*Purpureomyceskhaoyaiensis*	BCC 14290	Lepidoptera larva	JN049869	KX983469	KX983463	KX983458	NA	KX983466	Hywel-Jones (1994)
*Purpureomyceskhaoyaiensis*	BCC 44287	Lepidoptera larva	NA	KX983470	KX983464	KX983459	NA	KX983467	Hywel-Jones (1994)
*Purpureomycespyriformis*	BCC 85074^T^	Lepidoptera larva	MN781929	NA	MN781873	MN781730	MN781775	MN781821	Mongkolsamrit et al. (2020)
*Purpureomycespyriformis*	BCC 85348	Lepidoptera larva	MN781927	NA	MN781871	MN781728	MN781773	MN781820	Mongkolsamrit et al. (2020)
*Samsoniellaasiatica*	YFCC 869^T^	Lepidoptera pupa	OQ476473	OQ476497	OQ476505	OQ506153	OQ506195	OQ506187	Wang et al. (2023)
*Samsoniellahepiali*	YFCC 868	Hepialidae pupa	OQ476484	OQ476502	OQ476510	OQ506158	OQ506200	OQ506192	Wang et al. (2023)
*Sungiayongmunensis*	EFCC 2131^T^	Lepidoptera	JN049856	EF468977	EF468833	EF468770	EF468876	KJ398690	Sung et al. (2007a)
*Sungiayongmunensis*	EFCC 2135	Lepidoptera	NA	EF468979	EF468834	EF468769	EF468877	NA	Sung et al. (2007a)
*Tolypocladiumcapitatum*	NBRC 100997	Fungi	NA	JN941740	JN941401	AB968597	JN992474	AB968558	Schoch et al. (2012); Ban et al. (2015)
*Tolypocladiumcapitatum*	NBRC 106325	Fungi	NA	JN941739	JN941402	AB968598	JN992473	AB968559	Schoch et al. (2012); Ban et al. (2015)
*Tolypocladiumcylindrosporum*	ARSEF 2920^T^	Soil	NA	NA	MH871712	MG228390	MG228384	MG228387	Montalva et al. (2019)
*Tolypocladiumcylindrosporum*	YFCC 1805001	Soil	NA	MK984565	MK984577	MK984569	MK984584	MK984573	Weiser et al. (1991)
*Tolypocladiumpseudoalbum*	YFCC 876	Soil	NA	OP207718	OP207738	OP223152	OP223130	OP223140	Dong et al. (2022)
*Tolypocladiumpseudoalbum*	YFCC 875^T^	Soil	NA	OP207717	OP207737	OP223151	OP223129	OP223139	Dong et al. (2022)
*Yosiokobayasiakusanagiensis*	TNS F18494	Lepidoptera	JN049873	JF415954	JF415972	JF416014	JN049890	NA	Kepler et al. (2012a)
*Yosiokobayasiakusanagiensis*	BUM 1307	Lepidoptera	OM955151	OM951246	OM951251	OM988199	NA	OM988191	Sung et al. (2007b)

Boldface: data generated in this study; ^T^: ex-type culture. Culture collection acronyms: **ARSEF**: Agricultural Research Service Collection of Entomopathogenic Fungal Cultures; **BCC**: BIOTEC Culture Collection Laboratory; **BPI**: U.S. National Fungus Collections; **CBS**: the culture collection of the Westerdijk Fungal Biodiversity Institute; **CCTCCM**: China Center for Type Culture Collection and Microorganism; **EFCC**: Epping Forest Conservation Center; **GMB**: Guizhou Medical University Herbarium; **GZUHSB**: Guizhou University of Humanities, Science and Technology, Specimen Bank; **HKAS**: Herbarium of Cryptogamic Kunming Institute of Botany Academia Sinica; **IMI**: CABI Bioscience UK Center; **JCM**: Japan Collection of Microorganisms; **NBRC**: National Institute of Technology and Evaluation; **NHJ**: National Herbarium of Japan; **OSC**: Culture Collection of Oregon State University; **TNSF**: National Museum of Nature and Science; **YFCC**: Yunnan Fungal Culture Collection of Yunnan University; **YHH**: Yunnan Herbal Herbarium of Yunnan University.

## ﻿Results

### ﻿Phylogenetic analyses

**Figure 1.**
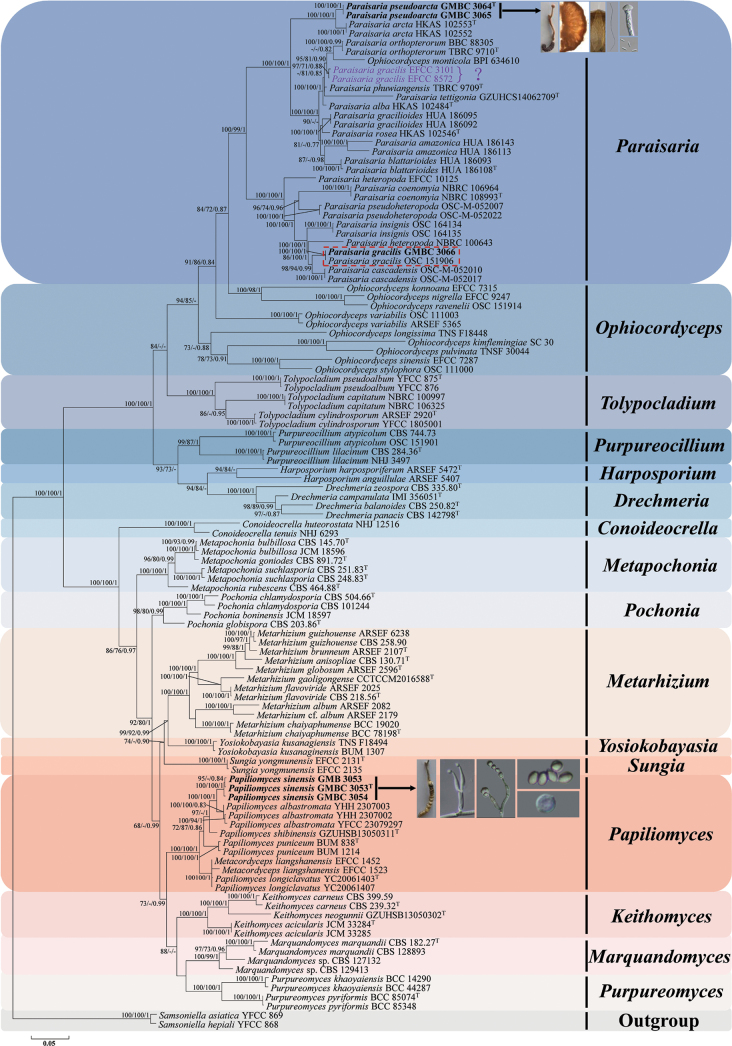
Phylogenetic tree based on combined partial nrSSU + ITS + nrLSU + *tef-1α* + *rpb1* + *rpb2* sequences showing the relationship of two new species of Lepidoptera from China with other species. Numbers at the branches indicate support values (IQ-TREE-BS/RAxML-BS/BI-PP) above 70%/70%/0.7. Ex-type materials are marked with “T.” Materials in bold type are those analyzed in this study.

The combined six-locus dataset contained 6,284 base pairs (bp) of sequences after alignment, including 1,631 bp for nrSSU, 787 bp for ITS, 959 bp for nrLSU, 992 bp for *tef-1α*, 759 bp for *rpb1*, and 1,156 bp for *rpb2*. Phylogenetic analyses based on the combined six-locus sequences from 113 fungal taxa confirmed the presence and positions of *Pap.sinensis* and *Par.pseudoarcta* within Clavicipitaceae and Ophiocordycipitaceae, respectively. Seventeen well-supported clades were recognized, which accommodate species of the genera *Conoideocrella*, *Drechmeria*, *Harposporium*, *Keithomyces*, *Marquandomyces*, *Metapochonia*, *Metarhizium*, *Ophiocordyceps*, *Papiliomyces*, *Paraisaria*, *Pochonia*, *Purpureocillium*, *Purpureomyces*, *Samsoniella*, *Sungia*, *Tolypocladium*, and *Yosiokobayasia* (Fig. 1). The phylogenetic analyses also resolved most *Papiliomyces* and *Paraisaria* lineages in separate terminal branches. It was proposed that one specimen and two strains (GMB 3053, GMBC 3053, and GMBC 3054), which formed a distinct lineage and had a close relationship with *Pap.albostromaticus*, might be a new species in the genus *Papiliomyces* (Clavicipitaceae), named *Pap.sinensis* (Fig. 1). Our analyses also revealed that the newly discovered species, *Par.pseudoarcta* (GMBC 3064 and GMBC 3065), were phylogenetically clustered with *Par.arcta* but clearly distinguished from the latter by forming a strongly supported clade in the genus *Paraisaria* (Ophiocordycipitaceae).

Despite differing topologies between individual loci (nrSSU, ITS, nrLSU, *tef-1α*, *rpb1*, and *rpb2*), the newly proposed species were consistently resolved as distinct clades from other known species. Some novel species consistently recovered sister relationships with particular known species across all loci. For example, the newly discovered species *Par.pseudoarcta* had a close genetic relationship with *Par.arcta*. These two species were distinguished as separate taxa with strong support from nrSSU, ITS, nrLSU, *tef-1α*, *rpb1*, and *rpb2* datasets (Suppl. material 1: figs S1–S6). Meanwhile, the new species *Pap.sinensis* was resolved as sister to *Pap.albostromaticus*, with significant bootstrap support from ITS, nrLSU, *rpb1*, and *rpb2* phylogenetic analyses (Suppl. material 1: figs S2, S3, S5, S6).

### ﻿Taxonomy

#### ﻿ Papiliomyces sinensis

Taxon classificationFungiHypocrealesClavicipitaceae

H. Chen & Y. Wang sp. nov.

7B3D059A-CABB-5A44-B004-753C05710F9F

857553

Fig. 2

##### Etymology.

Named after China (Guizhou and Yunnan province), where the species is distributed.

##### Type.

China • Guizhou Province, Shibing County, Yuntai Mountain Scenic Area (27°7'N, 108°7'E, alt. 1066 m), on a *Napialus* sp. buried in forest soil, May 2024, collected by Yao Wang (holotype: GMB 3053; ex-type GMBC 3053).

##### Description.

**Sexual morph: *Stroma*** solitary, fleshy, clavate, gray to earthy yellow, arising from the head of host, 51.3–85.7 × 3.1–3.5 (X̄ = 68.5 × 3.2, n = 5) mm. Perithecia, asci, and ascospores not observed. **Asexual morph**: Paecilomyces-like. Colonies on PDA slow-growing, up to 18 mm diam. in 14 days at 25 °C, white to gray, cottony with raised mycelial density at the center, generating several concentric rings at the edge, reverse yellowish to brown. ***Hyphae*** hyaline, septate, branched, smooth-walled, 0.9–2.2 µm wide. ***Conidiophores*** smooth-walled, cylindrical, mononematous, erect, aseptate, 18.1–41.3 × 0.8–2.0 (X̄ = 26.8 × 1.3, n = 30) µm. ***Phialides*** verticillate, in whorls of two to five, usually solitary on hyphae, basal portion cylindrical to narrowly lageniform, tapering gradually toward the apex, 10.1–26.9 × 0.9–3.3 µm (X̄ = 20.8 × 2.4, n = 30). ***Conidia*** in long chains, echinulate (visible under high magnification), globose, ellipsoidal, or ovoid, one-celled, 2.5–4.2 × 2.1–4.1 (X̄ = 3.5 × 2.8, n = 50) µm.

**Figure 2.**
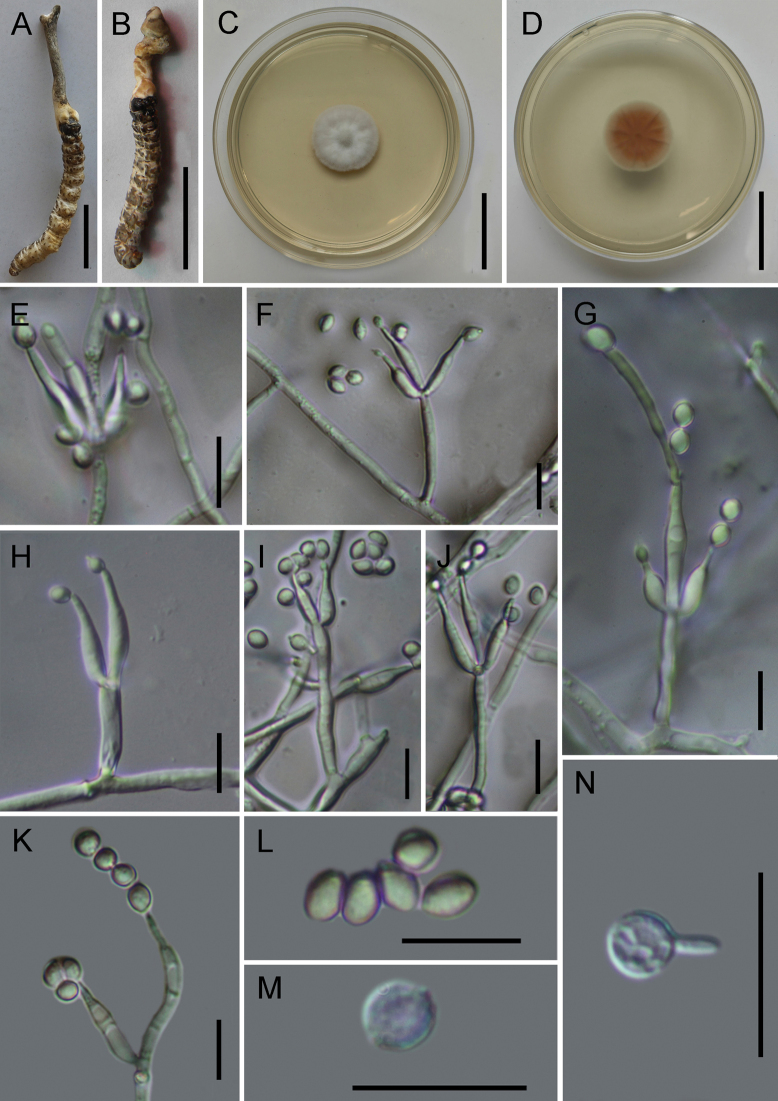
*Papiliomycessinensis***A, B** fungus on the host of *Napialus* sp. **C, D** culture character on PDA medium **E**–**K** conidiophores, phialides, and conidia **L** conidia **M**, **N** chlamydospores. Scale bars: 20 mm **(A**, **B)**; 15 mm **(C**, **D)**; 10 µm **(E**–**N)**.

##### Host.

Larva of *Napialus* sp. (Lepidoptera, Hepialidae).

##### Habitat.

In the soil of evergreen broadleaf forests and evergreen defoliated broadleaf mixed forests.

##### Distribution.

Guizhou and Yunnan Provinces, China.

##### Other material examined.

China • Yunnan Province, Shuifu County, Tongluoba National Forest Park (28°26'N, 104°8'E, alt. 1462 m), on larvae of *Napialus* sp. buried in forest soil, May 2024, collected by Yao Wang (GMB 3054, GMB 3076–GMB 3079; living cultures: GMBC 3054, GMBC 3076–GMBC 3079).

##### Notes.

*Papiliomycessinensis* phylogenetically clusters with *Pap.albostromaticus*, *Pap.shibinensis*, and *Pap.puniceum* but is distinguished from these three by forming a separate clade in this group (Fig. 1; 100%/100%/1). This species is morphologically closest to *Pap.albostromaticus*, having fleshy clavate stromata with gray color and *Paecilomyces*-like asexual conidiogenous structure. However, it is easily distinguished by its larger stromata (51.3–85.7 × 3.1–3.5 mm vs. 37.0–58.0 × 2.5–3.0 mm), longer phialides [10.1–26.9 (X̄ = 20.8) µm vs. 9.8–24.3 (X̄ = 16.2) µm] from *Pap.albostromaticus*, and spinulose conidia (Chen et al. 2024).

#### ﻿ Paraisaria pseudoarcta

Taxon classificationFungiHypocrealesOphiocordycipitaceae

Y. Wang & H. Chen sp. nov.

BF7B078D-EC2F-5E63-837E-B578276A3C4F

857554

Fig. 3

##### Etymology.

Referring to macromorphological resemblance of *Par.arcta*, but *Par.pseudoarcta* is phylogenetically distinct.

##### Type.

China • Yunnan Province, Lancang Lagu Autonomous County (22°32'N, 99°54'E, alt. 1176 m), on a Lepidopteran larva buried in forest soil, Jun 2024, collected by Yao Wang (holotype: GMB 3064; ex-type GMBC 3064).

##### Description.

**Sexual morph: *Stroma*** capitate, solitary, arising from the head of host, 43–51 mm long. ***Fertile head*** globose to subglobose, white to light brown, constricted at the center, with sticky and crystal-like substance on the surface, 4.6–4.8 × 4.8–4.9 mm. Stipe fleshy, white to light brown, slightly flexuous, 46.1–38.4 × 3.8–3.5 mm. ***Perithecia*** completely immersed, ampulliform to ellipsoidal, 776–979 × 252–339 (X̄= 876 × 289, n = 30) μm. ***Asci*** hyaline, narrow cylindrical, tapering toward the base, 8-spored, with thickened cap, 306–379 × 3.5–6.2 (X̄= 344 × 4.7, n = 30) μm. ***Apical cap*** 7.8–8.0 × 3.3–4.6 (X̄= 7.9 × 4.0, n = 20) μm. ***Ascospores*** hyaline, narrow filiform, equal to the asci in length, when mature, breaking into 64 cylindrical secondary ascospores. ***Secondary ascospores*** hyaline, smooth, one-celled, cylindrical, straight, 5.6–8.3 × 1.7–3.1 (X̄= 7.3 × 2.3, n = 30) μm. **Asexual morph**: Undetermined.

**Figure 3.**
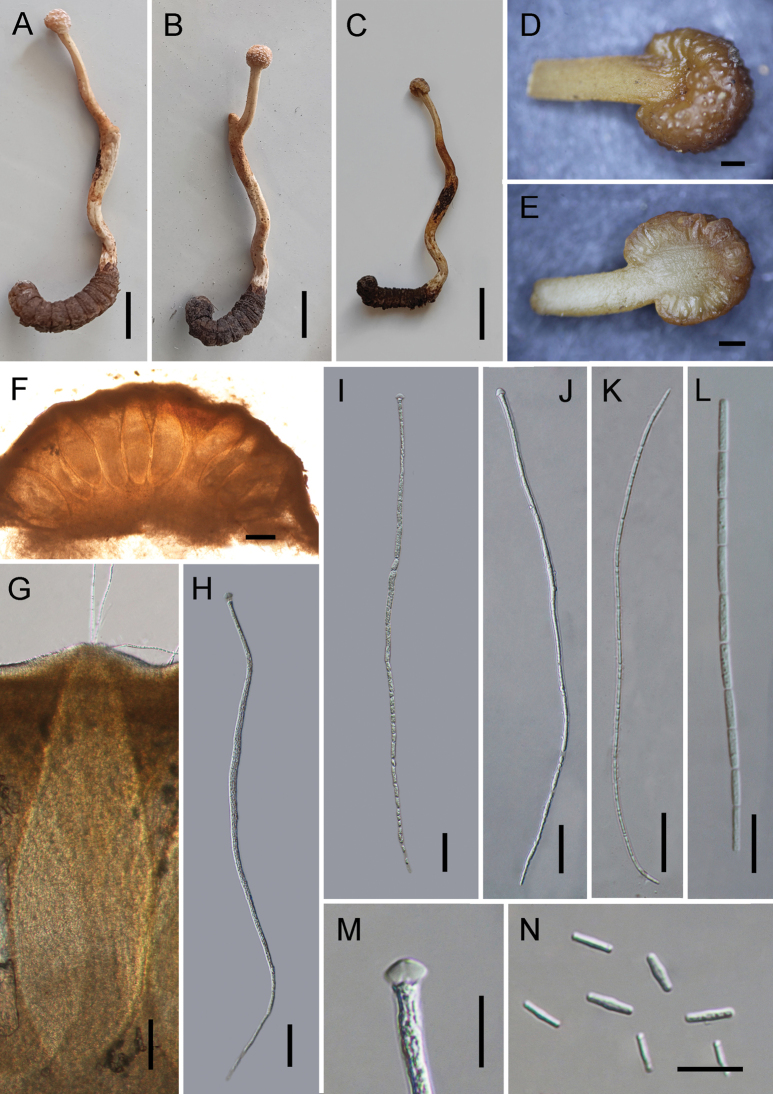
*Paraisariapseudoarcta***A**–**C** stromata emerging from host mouth **D** fertile part of stroma **E** cross section of fertile part showing arrangement of perithecia **F**, **G** perithecia **H**–**K** asci **L** ascospore **M** ascus cap **N** secondary ascospores. Scale bars: 10 mm **(A**–**C)**; 1 mm **(D**, **E)**; 100 μm **(F**, **G)**; 30 μm **(H**–**N)**.

##### Host.

Larva of Lepidoptera.

##### Habitat.

In the soil of evergreen broadleaf forests.

##### Distribution.

At present, it is known only in Lancang Lahu Autonomous County, Yunnan Province, China.

##### Other material examined.

China • Yunnan Province, Lancang Lagu Autonomous County (22°32'N, 99°54'E, alt. 1180 m), on Lepidopteran larvae buried in forest soil, Jun 2024, collected by Yao Wang (GMB 3065, GMB 3075; living culture: GMBC 3065).

##### Notes.

Morphologically, *Par.pseudoarcta* resembles the phylogenetic sister species *Par.arcta*. They were found to be parasitic on lepidopteran larvae, and they are easily recognized by having white and constricted fertile heads. However, our morphological analysis showed that *Par.pseudoarcta* and *Par.arcta* differed significantly in the size of their stromata and secondary ascospores. *Paraisariapseudoarcta* usually has long stromata (43–51 mm) and large secondary ascospores (5.6–8.3 × 1.7–3.1 μm), while *Par.arcta* has short stromata (16 mm) and small secondary ascospores (2.6–4.2 × 0.5–1.3 μm) (Table 3). In addition, molecular phylogenetic analyses indicated that they are distinct species (Fig. 1; 100%/100%/1).

**Table 3.** Morphological comparison between new species and related species.

Species	Stromata (mm)	Fertile head (mm)	Perithecia (µm)	Asci (μm)	Part-spores (μm)	Phialides (μm)	Conidia (μm)	References
*Papiliomycesalbastromata*	Clavate, mostly solitary, rarely multiple or branched, white, 37.0–58.0 × 2.5–3.0	Cylindrical, black to grayish white, 6.5–7.4 × 1.5–2.8	Completely immersed, long ovoid or teardrop, 236.9–365.6 × 76.8–122.7	Cylindrical, 8-spored		9.8–24.3 × 1.5–3.1	Ellipse or oval, smooth, 3.2–4.5 × 2.7–4.1	Chen et al. (2024)
*Papiliomyceslongiclavatus*	Clavate, solitary, greyish to light yellow, 40–60 × 3–5	Cylindrical, grey white to grey black, 15–21 × 4–6	Immersed, flask-shaped, 320–580 × 110–230	Narrowly cylindrical, 8-spored, 140–230 × 5–7	Cylindrical, 2–9 × 1–2	Two types of phialides, α-phialides, 13–24 × 1–2; β-phialides, 28–45 × 1–2	Two types of conidia, smooth, α-conidia, subglobose, 3–5 × 1–3; β-conidia, fusiform, 6–10 × 1–3	Zhang et al. (2023)
*Papiliomycespuniceum*	Clavate, solitary, red, 21.5 × 3.9	Not observed	Not observed	Not observed	Not observed	A little swollen base, slender top, 7.8–16.5 × 1.1–1.8	Echinulate, spherical, 3.0–5.9	Chen et al. (2023)
*Papiliomycesshibinensis*	Clavate, solitary, white to faint yellow, 42 × 2–3	Cylindrical or obtuse, faint yellow, 18–22 × 2–3	Completely immersed, elongated or ampulliform, 400–475 × 135–195	Cylindrical, 8-spored, 130–200 × 5.1–8.3	Not breaking into secondary ascospores			Wen et al. (2015)
***Papiliomycessinensis***	**Clavate, solitary, gray to earthy yellow, 51.3–85.7 × 3.1–3.5**	**Not observed**	**Not observed**	**Not observed**	**Not observed**	**10.1–26.9 × 0.9–3.3**	**Echinulate, globose, ellipsoidal or ovoid, 2.5–4.2 × 2.1–4.1**	**This study**
*Paraisariaarcta*	Solitary, 16 long	Subglobose with constriction at center, white, 2 × 3	Completely immersed, ampulliform to ellipsoidal, 230–530 × 70–180	Narrowly cylindrical, 8-spored, 100–180 × 2–4	Cylindrical, 2.6–4.2 × 0.5–1.3			Wei et al. (2021)
***Paraisariapseudoarcta***	**Solitary, 43–51 long**	**Globose to subglobose with constriction at center, white to light brown, 4.6–4.8 × 4.8–4.9**	**Completely immersed, ampulliform to ellipsoidal, 776–979 × 252–339**	**Narrowly cylindrical, 8-spored, 306–379 × 3.5–6.2**	**Cylindrical, 5.6–8.3 × 1.7–3.1**	**Not observed**	**Not observed**	**This study**

## ﻿Discussion

Two new species, *Papiliomycessinensis* (Ophiocordycipitaceae) and *Paraisariapseudoarcta* (Clavicipitaceae), were discovered through our taxonomic investigation. Morphological observations revealed sufficient phenotypic differences to justify their classification as distinct taxa.

Within the genus *Papiliomyces*, *Pap.sinensis* was newly described and shown to form a well-supported clade closely related to *Pap.albostromaticus*, *Pap.longiclavatus*, *Pap.puniceum*, and *Pap.shibinensis*, based on a six-locus phylogeny. Although morphologically similar to these congeners, *Pap.sinensis* can be distinguished by its gray to earthy-yellow stromata (Chen et al. 2023; Zhang et al. 2023; Chen et al. 2024).

Notably, *Pap.sinensis*, like several related species, was collected from larvae of Hepialidae (Chen et al. 2024). The habitats of Hepialidae larvae—such as alpine meadows, shrublands, and forest soils—provide favorable ecological conditions for the growth of entomopathogenic fungi. By utilizing the resources and spatial niches offered by the larvae, these fungi have adapted to thrive within the Hepialidae larval environment, facilitating their reproduction and dispersal. At the same time, they exhibit distinct ecological niches and growth patterns. Although *Metarhiziumlymantriidae* has not been reported to parasitize Hepialidae larvae specifically, *Metarhiziumlymantriidae* belongs to the same entomopathogenic fungal group and is known to infect a wide range of insect larvae, suggesting a possible host association with Hepialidae as well.

We also expanded the species diversity of the genus *Paraisaria* by describing a new species, *Par.pseudoarcta*. The sexual morph of this species resembles that of *Par.arcta*, featuring an erect or somewhat flexuous, cylindrical, hyaline, fleshy stipe terminating in a subglobose to globose fertile head with perithecia fully immersed in the tissue. The cylindrical asci possess a thickened apical crown, and the hyaline, multiseptate ascospores typically disarticulate into numerous cylindrical, truncated part-spores upon maturity. Nonetheless, *Par.pseudoarcta* can be distinguished by features such as the color of the fertile head, the number of stromata, and its host association. Members of this genus typically infect various insect developmental stages, including adults of Dictyoptera, Hymenoptera (ants), and Orthoptera; nymphs of Hemiptera and Orthoptera; and larvae of Coleoptera, Diptera, and Lepidoptera (Evans et al. 2010; Sanjuan et al. 2015; Mongkolsamrit et al. 2019). Although variations in color—ranging from white, pale pink, and pale rufous to chestnut, cinnamon buff, and dark brown—morphological variation in fertile head shape remains minimal. Eight asexual morphs have been described in this genus; however, the asexual stage of *Par.pseudoarcta* was not obtained in this study. Phylogenetic analyses placed *Par.pseudoarcta* in close relation to *Par.arcta*, further supporting its status as a distinct species. The new species differs from *Par.arcta* by having longer stromata and larger secondary ascospores in its sexual morph. Therefore, we recognize *Par.pseudoarcta* as a novel species.

This study also resolves a long-standing taxonomic issue concerning the misclassification of *Par.gracilis*. The nomenclatural status of *Par.gracilis* has long been problematic due to taxonomic synonymy. Greville (1823) initially placed the species in the family Xylariaceae as *Xylariagracilis*, and it was later formally described as *Cordycepsgracilis* by Durieu anf Montagne (1848), establishing the taxonomic foundation for *Par.gracilis* (Huang et al. 2019). Using molecular phylogenetic data, Sung et al. (2007a) reclassified two strains (EFCC 3101 and EFCC 8572) from *C.gracilis* to *Ophiocordycepsgracilis*. However, their *O.gracilis* diverged notably from the type species in host specificity, being isolated from coleopteran larvae rather than the lepidopteran hosts typical of *Par.gracilis*. Later, Mongkolsamrit et al. (2019) reassigned *O.gracilis* to *Paraisaria* within the Ophiocordycipitaceae. Quandt et al. (2014) included an additional strain, OSC 151906, in their phylogenetic reconstruction, which formed a separate clade from EFCC 3101 and EFCC 8572, suggesting the presence of an undescribed species—though no morphological comparison among these strains was made.

In our study, we collected specimens of *Par.gracilis* from the Altay region of Xinjiang and conducted detailed analyses. Morphological observations showed no significant differences from the teleomorphic and anamorphic characteristics previously reported by Samson and Brady (1983) and Gorbunova et al. (2011). Phylogenetic analysis placed our strain (GMBC 3066) in a distinct clade with OSC 151906, while EFCC 3101 and EFCC 8572 grouped with *Par.phuwiangensis* (Fig. 1). Despite topological inconsistencies in the single-locus trees (nrSSU, ITS, nrLSU, *tef-1α*, *rpb1*, and *rpb2*; Suppl. material 1: figs S1–S6), all datasets strongly supported species-level differentiation. Based on these results, we conclude that OSC 151906 and GMBC 3066 represent authentic *Par.gracilis*, while EFCC 3101 and EFCC 8572 warrant re-examination and comprehensive morphological redescription. Future research should also address unresolved nomenclatural and taxonomic ambiguities within this lineage.

## Supplementary Material

XML Treatment for Papiliomyces sinensis

XML Treatment for Paraisaria pseudoarcta

## ﻿Additional information

### Author ORCIDs

Xiangchun Shen https://orcid.org/0000-0002-4333-9106

Yao Wang https://orcid.org/0000-0002-1262-6700

### Data availability

All of the data that support the findings of this study are available in the main text or Supplementary Information.

## Supplementary materials

Supplementary material 1 Additional information Data type docx Explanation note **fig. S1.** Phylogenetic tree based on Maximum Likelihood (IQ-TREE) analysis from the nr SSU sequences. Statistical support values (≥70%) are shown at the nodes for ML boostrap support. Isolates in bold type are those analyzed in this stud. **fig. S2.** Phylogenetic tree based on Maximum Likelihood (IQ-TREE) analysis from the ITS sequences. Statistical support values (≥70%) are shown at the nodes for ML boostrap support. Isolates in bold type are those analyzed in this stud. **fig. S3.** Phylogenetic tree of based on Maximum Likelihood (IQ-TREE) analysis from the nr LSU sequences. Statistical support values (≥70%) are shown at the nodes for ML boostrap support. Isolates in bold type are those analyzed in this study. **fig. S4.** Phylogenetic tree based on Maximum Likelihood (IQ-TREE) analysis from the *TEF* sequences. Statistical support values (≥70%) are shown at the nodes for ML boostrap support. Isolates in bold type are those analyzed in this stud. **fig. S5.** Phylogenetic tree based on Maximum Likelihood (IQ-TREE) analysis from the *RPB1* sequences. Statistical support values (≥70%) are shown at the nodes for ML boostrap support. Isolates in bold type are those analyzed in this study. **fig. S6.** Phylogenetic tree based on Maximum Likelihood (IQ-TREE) analysis from the *RPB2* sequences. Statistical support values (≥70%) are shown at the nodes for ML boostrap support. Isolates in bold type are those analyzed in this study. https://binary.pensoft.net/file/1330665This dataset is made available under the Open Database License (http://opendatacommons.org/licenses/odbl/1.0/). The Open Database License (ODbL) is a license agreement intended to allow users to freely share, modify, and use this Dataset while maintaining this same freedom for others, provided that the original source and author(s) are credited.Hui Chen, Shabana Bibi, Ling Tao, Xiangchun Shen, Jun Zhao, Yueming Sun, Qirui Li, Dexiang Tang, Yao Wang
