# Supplementary material for: ﻿Papiliomycessinensis (Clavicipitaceae) and Paraisariapseudoarcta (Ophiocordycipitaceae), two new species parasitizing Lepidopteran insects from southwestern China
Source: MycoKeys. 2025 May 15;117:353–74. doi: 10.3897/mycokeys.117.150376 (PMC12099317; doi:10.3897/mycokeys.117.150376)
Supplement: Supplementary material 1 — Additional information [file mycokeys-117-353-s001.docx]

**
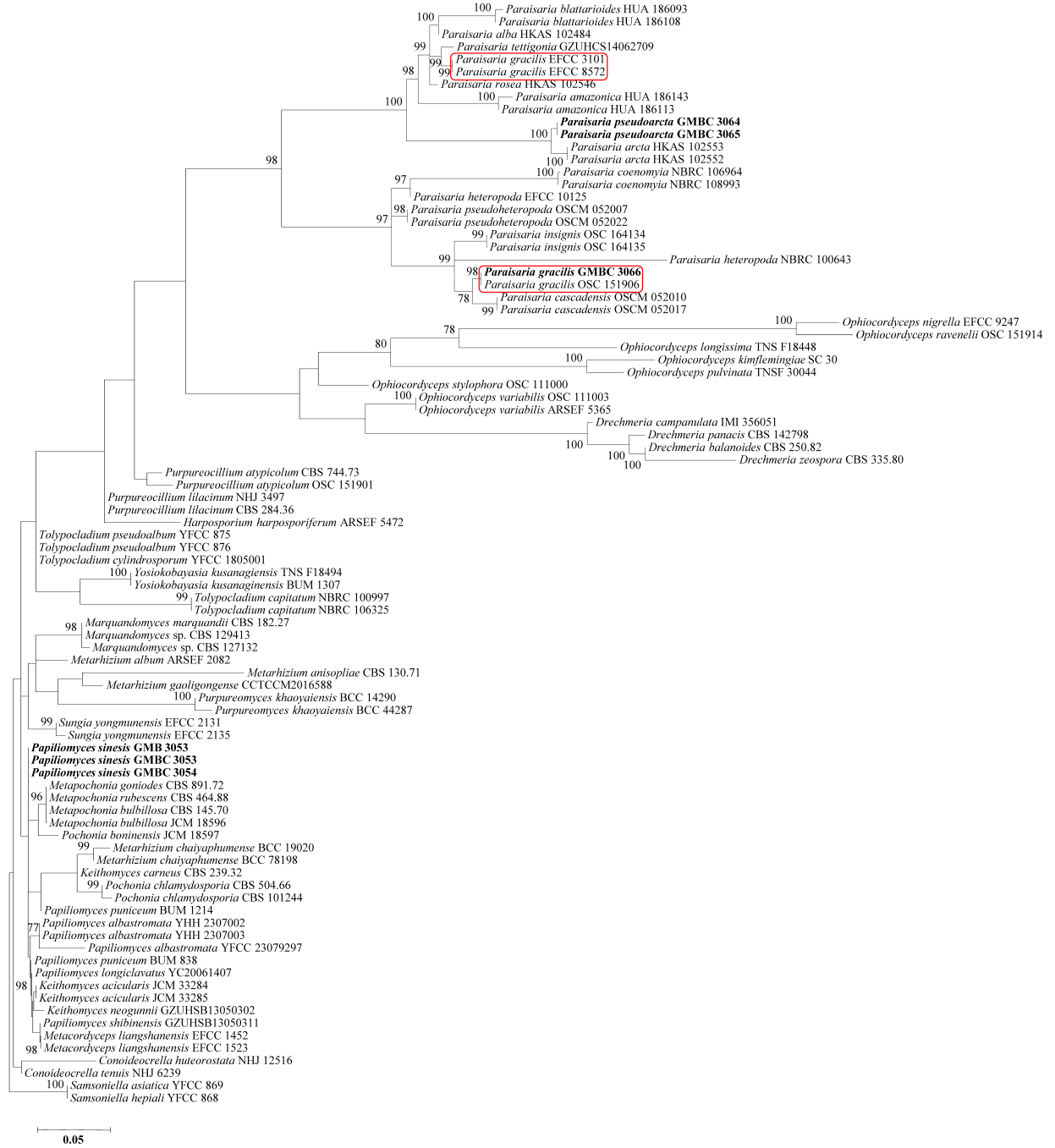
**

**Figure S1.** Phylogenetic tree based on Maximum Likelihood (IQ-TREE) analysis from the nr*SSU* sequences. Statistical support values (≥70%) are shown at the nodes for ML boostrap support. Isolates in bold type are those analyzed in this study.


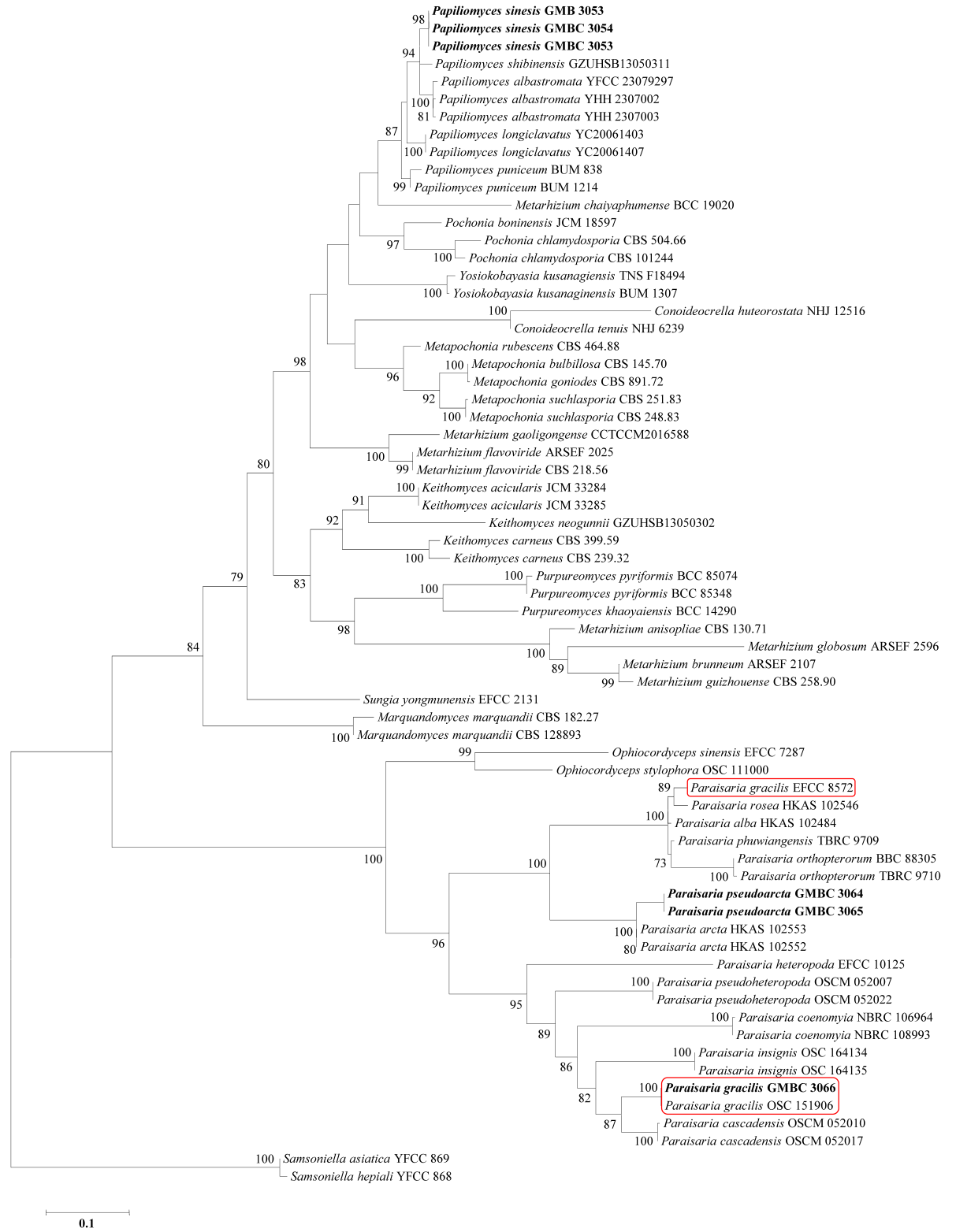


**Figure S2.** Phylogenetic tree based on Maximum Likelihood (IQ-TREE) analysis from the ITS sequences. Statistical support values (≥70%) are shown at the nodes for ML boostrap support. Isolates in bold type are those analyzed in this study.


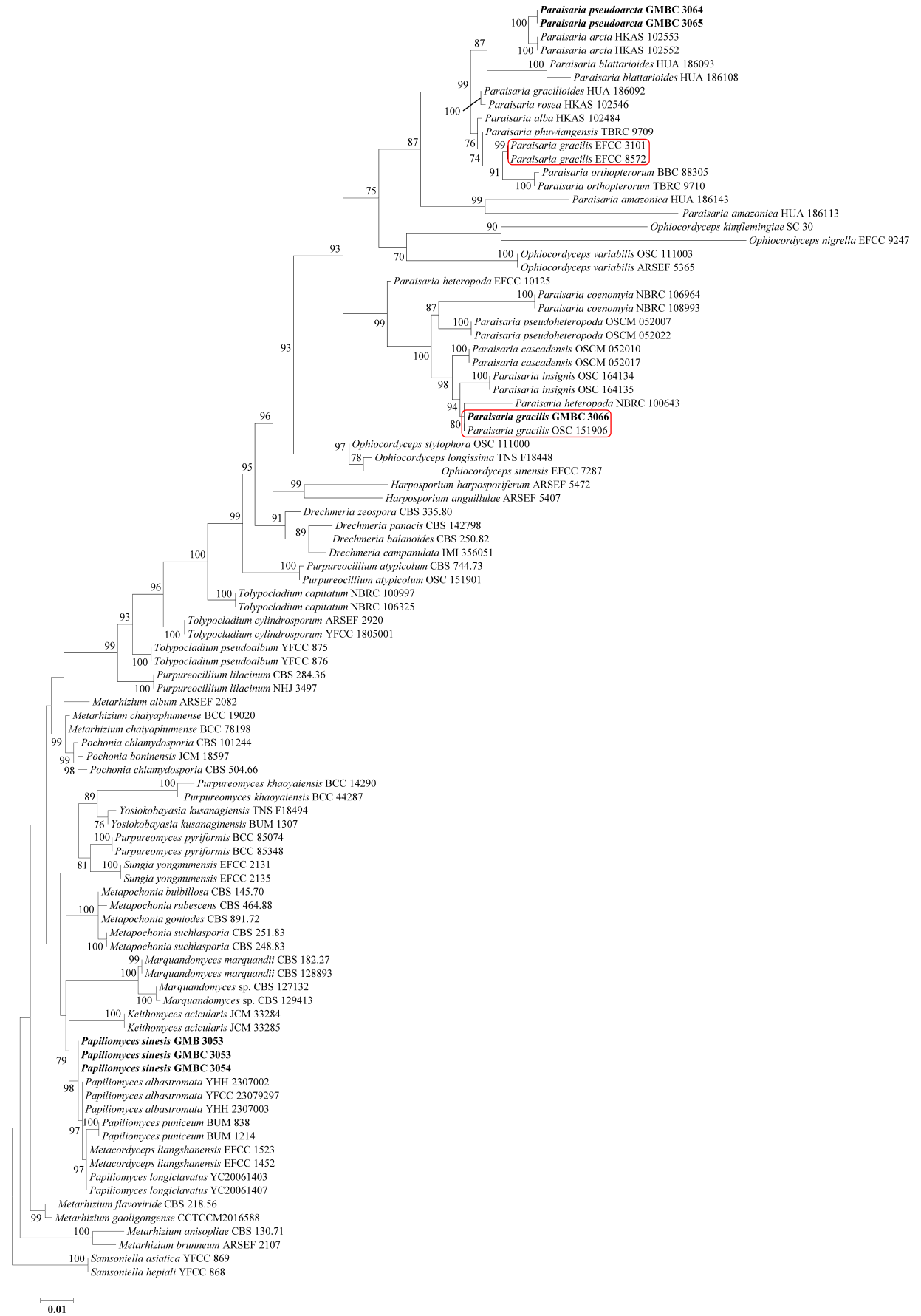


**Figure S3.** Phylogenetic tree of based on Maximum Likelihood (IQ-TREE) analysis from the nr*LSU* sequences. Statistical support values (≥70%) are shown at the nodes for ML boostrap support. Isolates in bold type are those analyzed in this study.


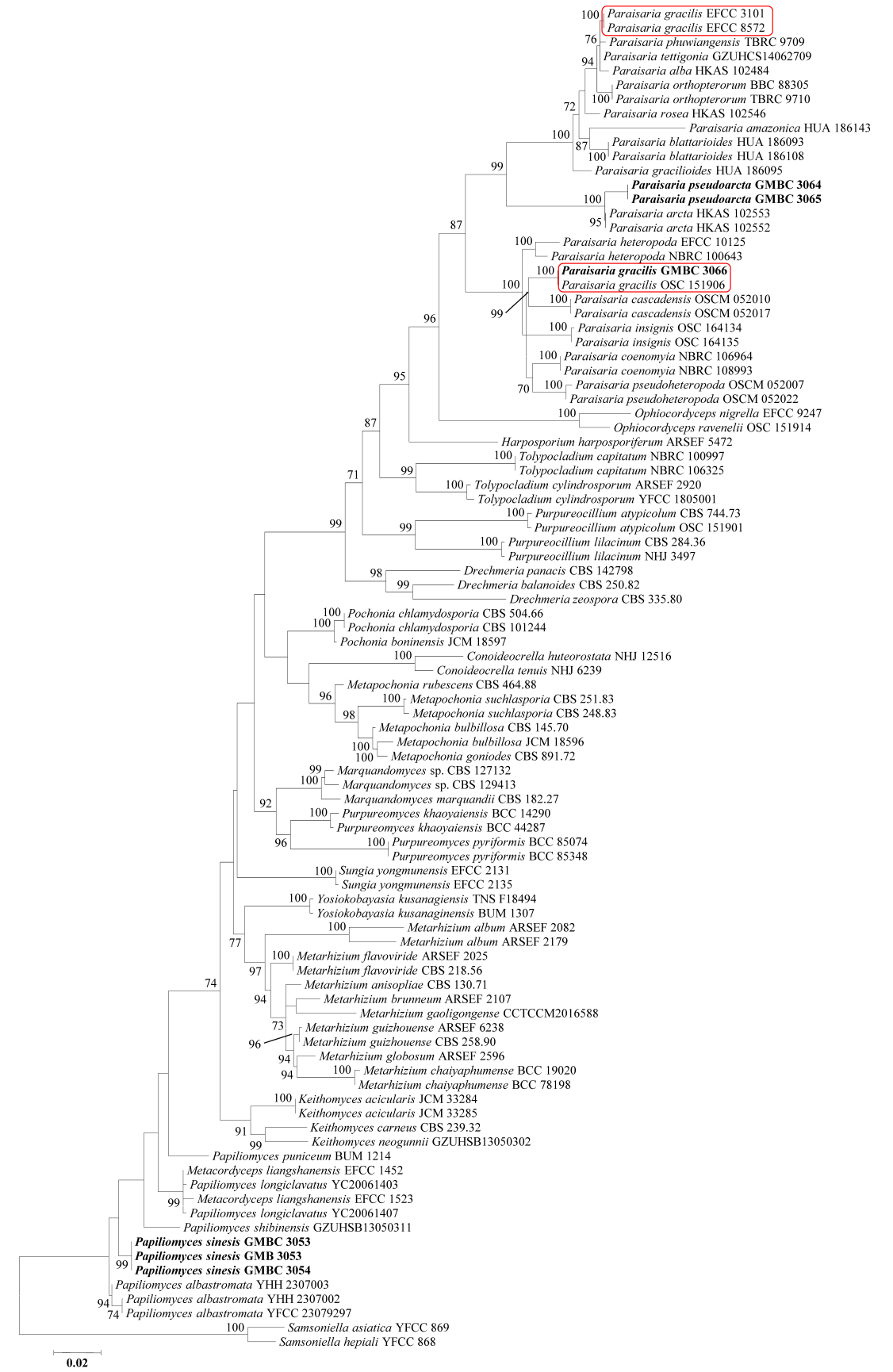


**Figure S4.** Phylogenetic tree based on Maximum Likelihood (IQ-TREE) analysis from the *TEF* sequences. Statistical support values (≥70%) are shown at the nodes for ML boostrap support. Isolates in bold type are those analyzed in this study.


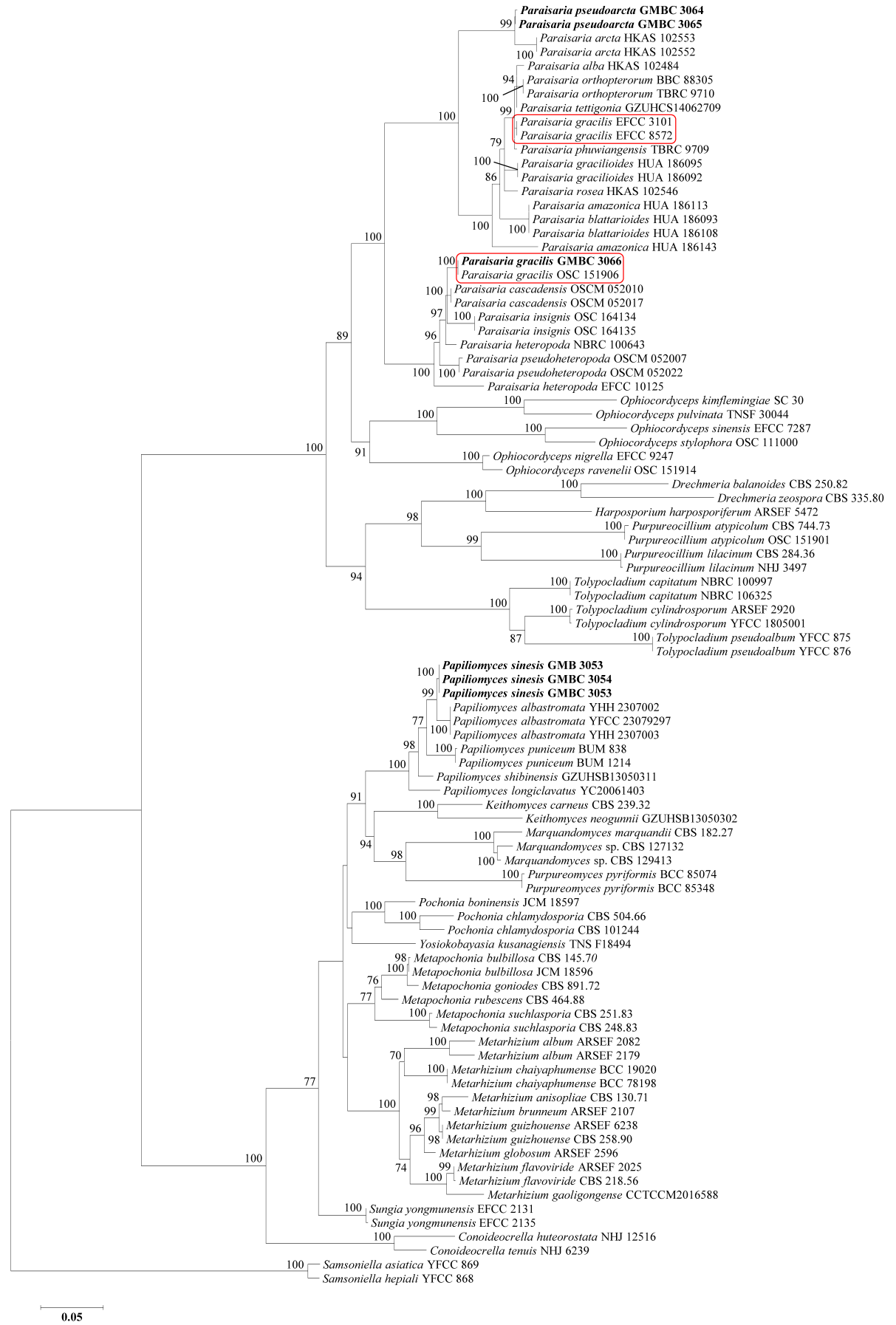


**Figure S5.** Phylogenetic tree based on Maximum Likelihood (IQ-TREE) analysis from the *RPB1* sequences. Statistical support values (≥70%) are shown at the nodes for ML boostrap support. Isolates in bold type are those analyzed in this study.


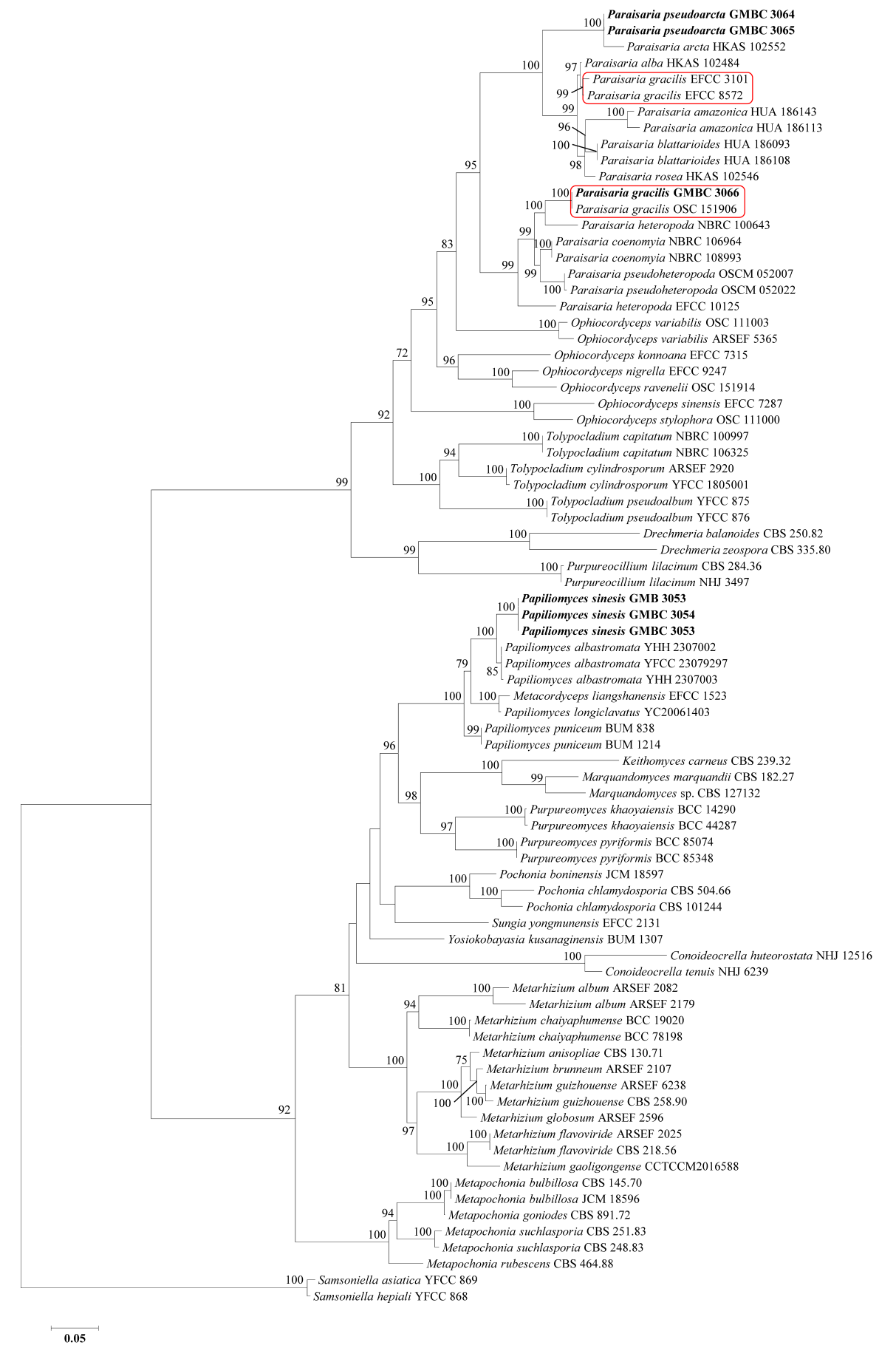


**Figure S6.** Phylogenetic tree based on Maximum Likelihood (IQ-TREE) analysis from the *RPB2* sequences. Statistical support values (≥70%) are shown at the nodes for ML boostrap support. Isolates in bold type are those analyzed in this study.
